# HPLC phenolic profile and induction of apoptosis by *Linum usitatissimum* extract in LNCaP cells by caspase3 and Bax pathways

**DOI:** 10.1186/s13568-020-01138-9

**Published:** 2020-11-10

**Authors:** Xin Zhou, Ningou Huang, Wenxin Chen, Tang Xiaoling, Behnam Mahdavi, Amir Raoofi, Davood Mahdian, Hadi Atabati

**Affiliations:** 1 Department of Urology, Beijing Tiantan Hospital,Capital Medical University, Beijing, 100070 China; 2 Department of Pharmacy, The Fourth Affiliated Hospital of Nanjing Medical University, Nanjing, 210031 Jiangsu China; 3 Department of Urology, Occupational Disease Hospital of Xinjiang Uygur Autonomous Region, Urumqi, 830000 Xinjiang China; 4 Jiangxi Research Institute of Traditional Chinese Medicine, Nanchang, 330046 Jiangxi China; 5 Department of Chemistry, Faculty of Science, Hakim Sabzevari University, 96179-76487 Sabzevar, Iran; 6 Cellular and Molecular Research Center, Department of Anatomy, Sabzevar University of Medical Sciences, Sabzevar, Iran; 7 Cellular and Molecular Research Center, Sabzevar University of Medical Sciences, Sabzevar, Iran

**Keywords:** *Linum usitatissimum*, Phenolic profile, Prostate cancer, Induction apoptosis, Bax, Caspase-3

## Abstract

*Linum usitatissimum* is a candidate as a remedy to treat prostate problems in some folklore medicines. In this study, we have reported the phenolic and flavonoid constituents, antioxidant activity, and potential of the plant extract against prostate cancer cells. The phenolic and flavonoid compound profile of the extract were established using HPLC analysis. While the total phenolic and flavonoid content (TPC and TFC) were analyzed using classic methods. The antioxidant activity of the extract was also evaluated. MTT assay and flow cytometry technique was used to evaluate antiproliferation activity and induction apoptosis of the plant extract on prostate cancer cells of LNCaP. We also evaluated the gene expression of Bax and caspase-3 using the real-time qPCR assay. HPLC result revealed that *L. usitatissimum* extract (LUE) was rich in phenolic acids such as gallic, ferulic, and vanillic acid with the amount of 3.56, 2.12, 1.24 μg/g extract respectively. 383.4 mg GAE/g and 47.1 mgRuE/g were calculated for total phenolic and flavonoid content. LUE exhibited radical scavenging activity with IC_50_ = 19.3 ± 1.1 µg/mL. LUE chelated ferrous ions with IC_50_ = 121.1 ± 1.3 µg/mL. LUE showed anti-proliferative activity on LNCaP cells with the IC_50_ values of 8.3, 6.3, and 5.4 μg/mL after 24, 48, and 72 h treatment. LUE also increased cell mortality by inducing apoptosis (15.3–29.8%). The real-time qPCR results exhibited an increase in gene expression of Bax and caspase-3. Our in vitro study demonstrates that *L. usitatissimum* can be considered as an effective agent to inhibit the growth and invasion the human prostate cancer cells.

## Introduction

Prostate cancer (PCa) is the most common form of cancer among males. But if detected in the early stages, because of the slow progression of the disease, the survival rates are high (Yoo et al. 2019; Chen et al. 2019)*.* The problem is typically observed in men with middle age or older (Yoon et al. 2016). The treatment decisions are chosen based on tumor aggressiveness (Abou-Hashem et al. 2019). Surgery, chemotherapy, or hormone therapy are common ways to treat PCa (Dasari et al. 2018b; Mirza et al. 2018), while natural plant products are candidates to treat PCa in some traditional medicine systems around the world. Black pepper, ginger, chili, and turmeric, due to their secondary metabolites, have been known as the plants with anticancer properties (Dasari et al. 2018b)*.* Hence, development in a novel natural therapeutic agent field is vital to improve the overall survival of people with PCa. On the other hand, dietary constituents are also critical probable risk factors in treating prostate cancer. In recent years, nutritional agents such as lycopene, vitamin C, and vitamin K, which possess anticancer activity, play an important role in various researches related to cancer problems (Dasari et al. 2018a).

*Linum usitatissimum* is known with the common name of flax or linseed. The plant is a member of the Linaceae family (Safarpoor et al. 2018). Flax is one of the most ancient crops with some specific in cloths and paper industries (Wei et al. 2018). Flaxseed is a valuable source of dietary fibers (Nwachukwu and Aluko 2018). The plant seeds are a source of carbohydrates, phenolic, flavonoids compounds, and lignans. Hemicellulose and cellulose are the main carbohydrates; ferulic, chlorogenic, gallic, and 4-hydroxybenzoic acids are the main phenolic compounds; secoisolariciresinol diglucoside is the main lignan, and herbacetin diglucoside is the main flavonoid compound in the plant (Herchi et al. 2016; Lazić et al. 2018; Garros et al. 2018; Fadzir et al. 2018). *L. usitatissimum* also contains fatty acids such as α-linolenic, linoleic, and oleic acid as well as mucilage vitamin B1, and vitamin A (Duygu and Yilmaz 2018). The plant is a good source of minerals such as magnesium, phosphorus, manganese, selenium, and zinc (Calado et al. 2018). Many reports are indicating that flaxseed possesses various bioactivities such as anticancer, anti-obesity, anti-diabetic activity, antiviral, antibacterial, anti-inflammatory antioxidant activity, and cardio-protective agent (Hu et al. 2019; Zhu and Li 2019a, b). Some researchers have proposed that flaxseed is a potential agent to reduce the proliferation of breast cancer (Calado et al. 2018), colon cancer (DeLuca et al. 2018), and skin cancer cells (Sharma et al. 2014).

In the texts related to traditional medicines, *L. usitatissimum* has been proposed to cure prostate problems (Azadbakht et al. 2019). Hence, in this study, we focus on evaluating the antiproliferation and induced apoptosis of the hydroalcoholic extract of *L. usitatissimum* on the human prostate cancer cells of LNCap. We have also studied the effect mechanism of the extract through the gene expression of Bax and caspase3.

## Materials and methods

### Preparation of plants extracts

*Linum usitatissimum* seeds were purchased from a medicinal plant store (Sabzevar, Iran). The seed was ground and macerated in ethanol:water (70:30) for 72 h. After filtration, the solvent was evaporated under reduced pressure using a Buchi evaporator (50 ºC). Finally, *L. usitatissimum* extract (Urhan et al. 2020) was dried in an oven at 50 ºC.

### HPLC analysis and identification of the main compounds

Extract at a concentration of 10 mg/mL in methanol was analyzed by HPLC (Waters 2695 (USA) and a PDA detector Waters 996 (USA) as described previously with some modification in the gradient system and flow rate (Gabr et al. 2018). The chromatographic assay was performed on a 15 cm × 4.6 mm with pre-column, Eurospher 100–5C_18_ analytical column provided by Waters (Sunfire) reversed-phase matrix (3.5 μm) (Waters). Elution was carried out in a gradient program with 0.5% (v/v) aqueous phosphoric acid (eluent A) and of 40% (v/v) aqueous acetonitrile (eluent B) with the flow-rate of 0.5 mL min^−1^. Peaks were monitored at wavelengths of 254, 278, 300, and 370 nm. The injection volume was 20 µL, and the temperature was maintained at 35 °C. The constituents were recognized by comparison of the retention time and UV–Vis. spectral reference data with those of the standard controls. The amounts of the various compounds were extrapolated from calibration standard curves. All standards were purchased from Sigma- Aldrich.

### Antioxidant activity assays

Determination of total phenolic content (TPC), total flavonoid content (TFC), radical scavenging activity (RSA), ferrous ion chelating (FIC) were carried out according to previous studies (Mahdavi et al. 2013; Hosseinpoor Mohsen Abadi et al. 2016). The chemicals for antioxidant assays were prepared from different companies as following:1,1-diphenyl-2-picrylhydrazyl (DPPH), α-tocopherol, 3-(2-pyridyl)-5,6-*bis*(4-phenylsulfonic acid)-1,2,4-triazine (ferrozine), butylated hydroxytoluene (BHT) were purchased from Sigma; ascorbic acid (Pallag et al. 2016), and EDTA from Merck; Na_2_CO_3_, AlCl_3_, FeSO_4_·7H2O, Folin-Ciocalteu’s reagent (FCR) from Fluka; gallic acid (GA) from Acros; and all solvents of analytical grade were obtained from Merck.

### Determination of total phenolic content (TPC)

A 0.5 mL of FCR (10% in distilled water) was added to 0.5 mL of the extract (100 µg/mL in methanol) and 1.5 mL of distilled water. After 5 min, two mL of Na_2_CO_3_ (5%) was added and shaken again. The mixture was kept in the dark for 2 h at room temperature. The absorbance was read at 760 nm using a Photonix Ar 2015 UV-Vis. instrument. The analyses were run in triplicates. TPC was measured as gallic acid equivalent (GAE), that is, mg of gallic acid equivalent per gram of extract (mg GAE/g extract).

### Determination of total flavonoid content (TFC)

A mixture of the plant extract (1 mL, 100 µg/mL) and a methanolic solution of AlCl_3_ (1 mL, 2%) was kept at room temperature for 30 min. Then, the mixture absorbance was read at 415 nm. The analyses were run in triplicates. TFC amount was obtained using a standard curve of rutin (10–160 µg/mL). TFC was expressed in mg of rutin equivalent per gram of dried extract (mg RuE/g extract).

### Determination of radical scavenging activity (RSA)

A 1.5 ml of methanolic solution of LUE (5–30 µg/mL) was added to 1 mL of DPPH (0.1 mM). The mixture was shaken and kept in the dark for 90 min at room temperature; the absorbance was read at 517 nm. Positive controls of butylated hydroxytoluene (BHT) and α-tocopherol (Toc) were used. All analyses were run in triplicates. The RSA was calculated using the following equation:

$$ {\text{RSA}}\% \, = \, \left[ {\left( {{\text{A}}_{{\text{c}}} - {\text{A}}_{{\text{s}}} } \right)/{\text{A}}_{{\text{c}}} } \right] \, \times {1}00 $$

where A_c_ is the absorbance of the control (DPPH solution without extract), and A_s_ is the absorbance of the extract (extract with DPPH solution).

### Ferrous ion chelating ability assay

First. A mixture of FeSO_4_ (50 µL, 2 mM), the plant extract solution in methanol (1 mL, 40–200 µg/mL), and distilled water (2 mL) was prepared. Then, ferrozine (100 µL, 5 mM) was added. The mixture was shaken and incubated at room temperature for 10 min. The absorbance was read at 562 nm. All evaluations were carried out in triplicates. EDTA (disodium salt) and ascorbic acid (AscA) were used as the positive controls. FIC for the plant extract was determined using the following equation:

$$ \% {\text{ Inhibition }} = \, \left[ {\left( {{\text{A}}_{{\text{c}}} {-}{\text{ A}}_{{\text{s}}} } \right)/{\text{A}}_{{\text{c}}} } \right] \, \times { 1}00 $$

where A_c_ is the absorbance of the control (contains FeSO_4_, ferrozine, and water), and A_s_ is the absorbance of the sample.

### MTT assay

The prostate cancer cell line (LNCaP) was purchased from Pastor Institute (Iran). Dulbecco’s modified Eagle’s medium (GIBCO, England) supplemented with 10% fetal bovine serum (GIBCO, England) and 5% penicillin (Sigma Aldrich, USA) was used. The cytotoxicity of LUE on LNCaP cells was studied using MTT assay according to a previous report with some modifications (Ni et al. 2019). Briefly, the cells were uniformly distributed (5 × 10^3 ^cells in each well) in a 96-wells plate and incubated at 37 °C with 5% CO_2,_ overnight. Then LUE at different concentrations (2–10 µg/mL) was added to the wells and incubated for 24, 48, and 72 h. Next, MTT (20 µl, 5 mg/mL in PBS) was added to the wells and incubated at 37 °C for 4 h. Finally, the formazan crystals were dissolved in dimethyl sulfoxide (DMSO) (100 µL) and the optical density was read at the wavelength of 570 nm and 630 (control wavelength) by a plate reader (Thermo Lab systems, Franklin, MA USA). IC_50_ (concentration of the extract that attained a 50% of mortality) of LUE was determined through Prism software. All treatments run in triplicate. In the MTT assay of the present study, DMSO was used as negative control and Docetaxel (0.1 µM) was the positive control.

### Cell proliferation

MTT assay was used to examine the cell proliferation. The cells in the density of 2 × 10^3^ cells/mL were plated in a 96 wells cell culture plate for 12 h. Next, various treatments were incubated for 48 h at 37 °C and 5% CO_2_. Then, 5 mg/mL of MTT powder (Sigma) was added to each well for 3 h. The supernatant of each well was removed. The formazan crystals were dissolved in DMSO (100μL) at room temperature. Finally, we used 200 μL of DMSO for each well. The absorption of various treatments were read in 570 and 630 nm references using an enzyme-linked immunosorbent assay (ELISA) Reader (Ni et al. 2019).

### Secretion of TNFα

The Rat inflammatory cytokine assay kit, Rat Kit V-Plex was used to measure the TNFα concentration.

### Flowcytometric analysis

A quantitative assessment of apoptosis was carried out using propidium iodide (PI) staining of small DNA fragments followed by flow cytometry. The assay was carried out according to a previous report (Kilinc et al. 2020). Briefly, LNCaP cells were cultured (1.5 × 10^5^ cells) with 10% FBS involved media and incubated for 24 h at 37 °C and 5% CO_2_. Next, the media was changed with serum-free media for 6 h and treated with LUE with different concentrations and times: 6, 8.3, and 10 µg/mL for 24 h; 4, 6.3, and 8 µg/mL for 48 h; and 4, 5.4, and 6 for 72 h. After incubation, floating and adherent cells were intake and incubated overnight with 750 μL of a hypotonic buffer consist of 50 mg/ml PI in sodium citrate (0.1%) with Triton X-100 (0.1%) at 4 °C in the dark. Next, flow cytometry was carried out using a flow cytometer (Becton Dickinson). A total of 1 × 10^4^ events were achieved with FACS and data were analyzed through flow Jo- V10 software.

### Real-Time Quantitative RT-PCR

Notably, qRT-PCR has been selected to evaluate the level of Bax and caspase3 expression. 500,000 of LNCaP cells were seeded in 6-well plates and incubated overnight. Then cells were exposed to LUE for 24, 48 and 72 h with IC_50_ doses. Furthermore, the total RNA has been derived from cell samples using the company guidelines for Trizol reagent. Then, cDNA has been synthesized according to the total RNA via a Prime-Script RT reagent kit with gDNA Eraser (Takara: Dalian). For reverse transcription-polymerase chain reaction (RT-PCR), the PCR reaction involved 35 cycles of denaturation for thirty seconds at 94 °C, an extension for thirty seconds at 72 °C, and Annealing for thirty seconds at 54 °C. Besides, PCR products for Bax and casp3 respectively have been 108 and 70 bp. Table 1 presents the primers. To obtain the real-time quantitative RT-PCR, we used the Fast-Start Universal SYBR Green Master (Roche: USA) over a Master cycle repeal plex 4 system to carry out the processes. Each reaction runs for three times. Finally, the comparative 2^−^ΔΔCt method has been used to determine mRNAs relative expression and then data normalized versus GAPDH.

**Table 1:** Primer sequences of Bax, Caspase-3, and GAPDH

Gene	Sequence	Primer sequence	Annealing	Product size (bp)
Bax	F R	TTCCGAGTGGCAGCTGAGATGTTT TGCTGGCAAAGTAGAAGAGGGCAA	54 °C × 30 s	108
Caspase-3	F R	ACTGGACTGTGGCATTGAGA GCACAAAGCGACTGGATGAA	54 °C × 30 s	70
GAPDH	F R	ATCTGACATGCTGCCTGGAG AAGGTTGGAAGATGGGAGTTGC	60 °C × 25 s	88

## Results

### HPLC analysis

**Fig. 1:**
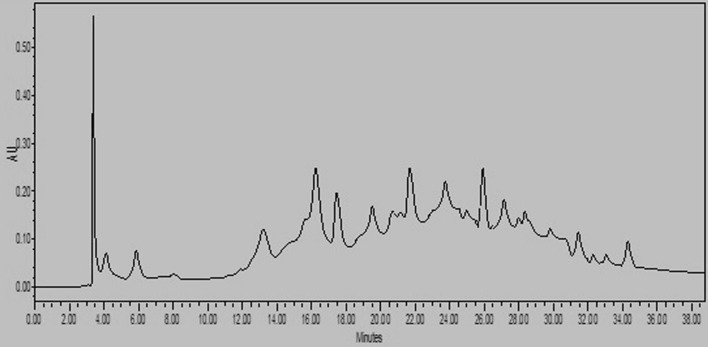
HPLC chromatogram of *Linum usitatissimum* Extract. Identification and quantification of compounds was done by comparing retention time and spectra of the peaks in the extract against that of the standard compounds

Figure 1 presents the HPLC chromatogram of LUE. The obtained results for the HPLC analysis are revealed in Table 2. Among the selected standards, including eight phenolic acids (gallic, vanillic, chlorogenic, caffeic, *p*-coumaric, sinapic, ferulic, and *trans*-*o*-hydroxy cinnamic acid) and two flavonoids (quercetin and rutin), LUE was rich in gallic, ferulic, and vanillic acid with the amount of 3.56, 2.12, 1.24 μmol/g extract respectively. According to our literature review, the presence of quercetin (0.16 μmol/g extract) and rutin (0.24 μmol/g extract) is reported for the first time.

**Table 2:** HPLC results of selected phenolics and flavonoids of *Linum usitatissimum* seeds extract

Compounds	Wavelength nm	RT (min.)	μmol/g extract	μg/g extract	Area
Phenolic acids					
Gallic acid	278	5.86	3.56	605.62	1,803,295
Vanillic acid	254	13.67	1.24	208.5	478,244
Chlorogenic acid	300	16.27	1.23	435.8	1,101,354
Caffeic acid	300	17.62	0.15	27.04	68,663
* p*-Coumaric acid	300	22.24	0.43	82.56	208,254
Sinapic acid	300	23.96	0.98	319.77	1,321,751
Ferulic acid	300	25.93	2.12	411.66	1,071,573
* trans*-*o*-Hydroxy cinnamic acid	254	31.42	0.52	85.36	186,843
Flavonoids					
Rutin	254	27.15	0.24	146.52	339,592
Myricetin	370	33.46	-	-	0
Quercetin	370	34.57	0.16	48.36	119,801

### Total phenolic content (TPC) and total flavonoid content (TFC)

TPC and TFC results of LUE are exhibited in Table 3. According to the results, 383.4 mg GAE/g was calculated for TPC of the extract; while, 47.1 mgRuE/g was measured for TFC of *L. usitatissimum* extract.

**Table 3:** Total phenolic content (TPC), total flavonoid content (TFC), DPPH radical scavenging activity (RSA), and ferrous ion chelating ability (FIC) of *Linum usitatissimum* extract

	TPC mg GAE/g extract	TFC (mgRuE/g extract)	RSA IC_50_ (μg/mL)	FIC IC_50_ (μg/mL)
LUE	383.4	47.1	19.3 ± 1.1	121.1 ± 1.3
BHT	–	–	17.4 ± 0.5	–
TOC	–	–	35.6 ± 0.6	–
EDTA	–	–	–	68.2 ± 1.2
AscA	–	–	–	1480.0 ± 3.2

Values are presented as means ± SD (n = 3)

### Radical scavenging activity (RSA) and ferrous ion Chelating (FIC)

The results are shown in Table 3. The extract with IC_50_ of 19.27 ± 1.1 µg/mL showed radical scavenging activity stronger than the positive control (α-tocopherol), but weaker than BHT. For FIC assay, LUE with IC_50_ of 121.01 ± 1.3 µg/mL was more active than ascorbic acid, while the ability of EGE to chelate ferrous ions was weaker than that of the positive control (EDTA).

### The effect of *Linum usitatissimum* seeds extracts on cell proliferation

**Fig. 2:**
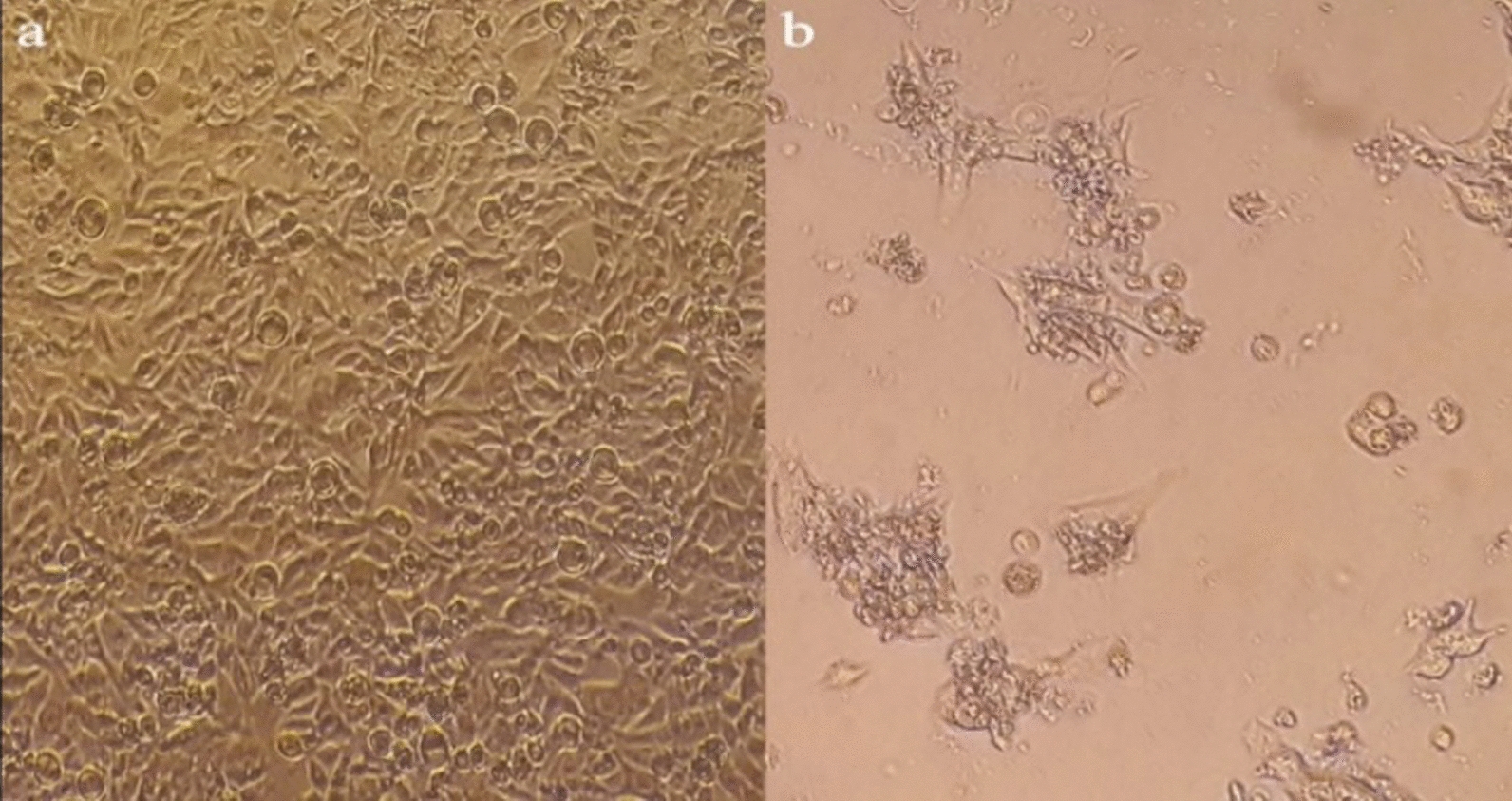
The morphology of human prostate cancer (LNCap cells) **a** before treatment with hydroalcoholic extract of *L. usitatissimum*, **b** after treatment with hydroalcoholic extract of *L. usitatissimum* (after 48 h)

Figure 2 shows the morphology of LNCaP cells. Figure 1a shows the morphology of the living prostate cancer cells of LNCaP before treatment with LUE. Figure 1b exhibited the cells after treatment with the extract after 48 h, as it can be seen, the morphology of the cells has changed and the extract inhibited the growth of the cells.

**Fig. 3:**
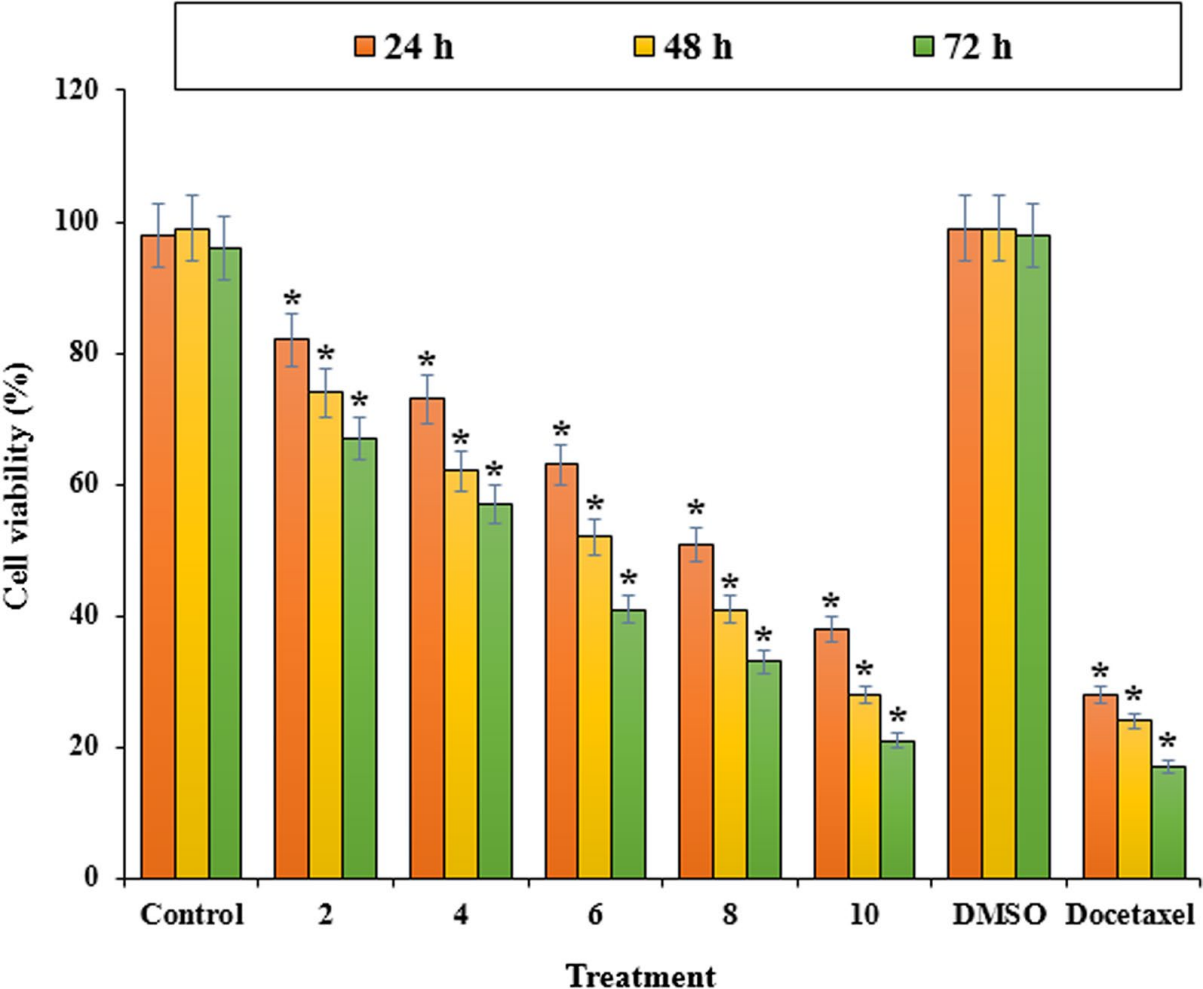
The effect of *L. usitatissimum* extract on the viability of LNCaP cells. The viability percentage was measured by MTT assay. LNCaP cells were treated with different concentrations of the extract for 24, 48, and 72 h. *Indicate the significant difference (*p* < 0.01) between experimental groups with control/DMSO groups

**Fig. 4:**
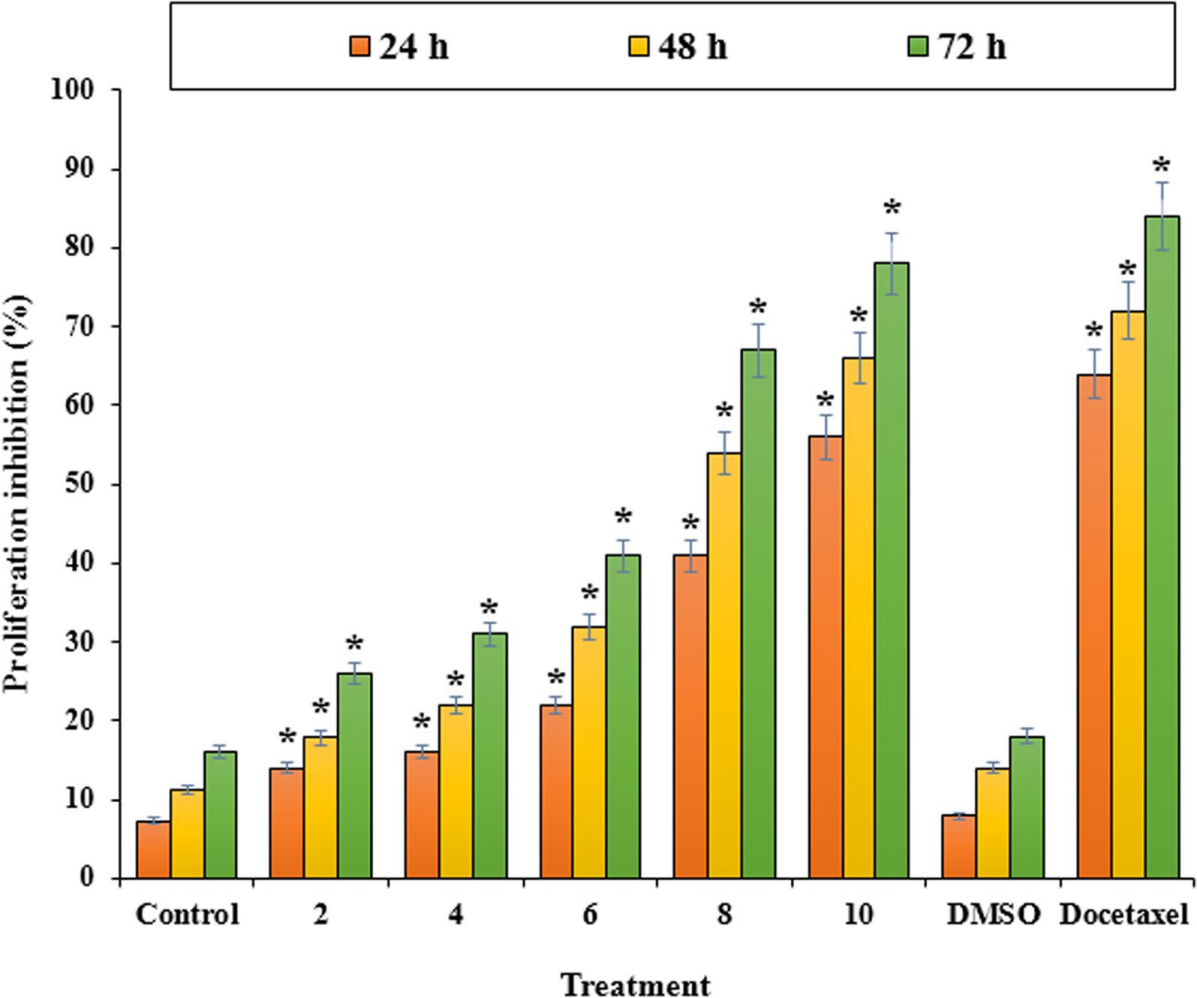
The effect of *L. usitatissimum* extract on the proliferation inhibition of LNCaP cells. The percentage was measured by MTT assay. LNCaP cells were treated with different concentrations of the extract for 24, 48, and 72 h. *Indicate the significant difference (*p* < 0.01) between experimental groups with control/DMSO groups

According to the result of the cytotoxic assay Fig. 3, LUE exhibited a concentration and time depending cytotoxic activity on LNCaP cells line at 24, 48, and 72 h. The extract showed a sufficient cytotoxic effect on LNCaP cells. The values of 8.3, 6.3, and 5.4 μg/mL were calculated for the extract IC_50_ for 24, 48, and 72 h treatment respectively. Besides, Fig. 4 indicates the excellent proliferation inhibition of LUE extract in the high doses compared with Docetaxel as a positive control.

### Detection of apoptosis by flow cytometry

**Fig. 5:**
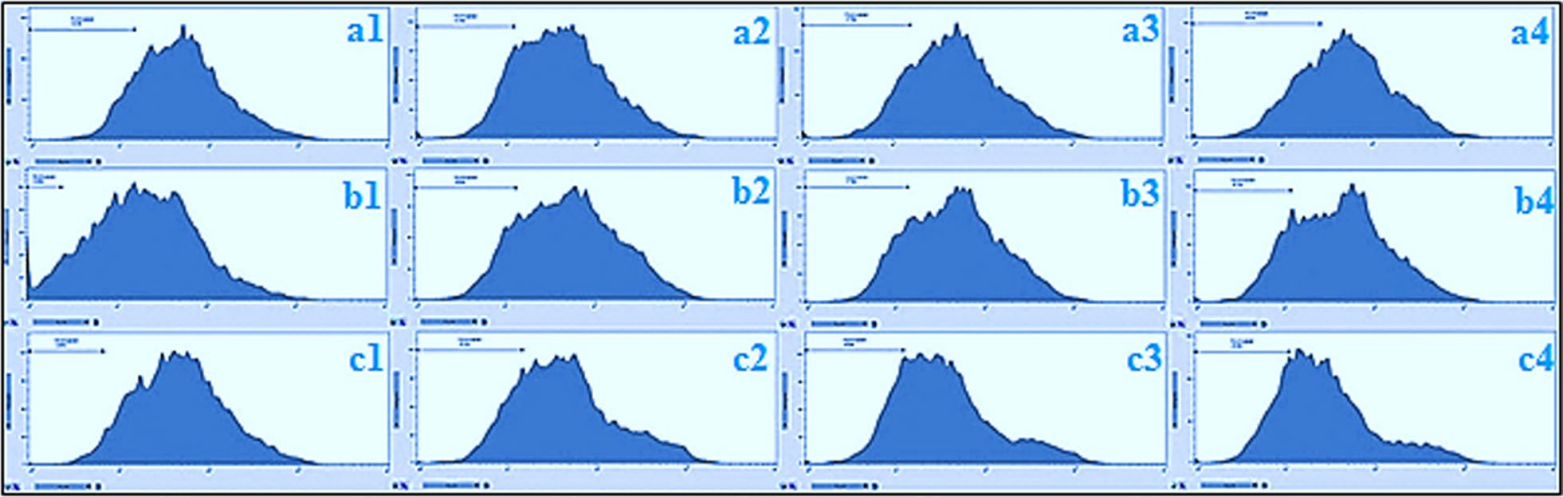
Flow cytometric evaluation of induced apoptosis using Propidium iodide staining by *L. usitatissimum* extract extract in LNCaP cells at different times and concentration.a1-4 after 24 h a1: control a2-4 concentrations of 6.0, 8.3, 10.0 μg/mL; b1-4 after 48 h b1: control, b2-4concentratoions of 4, 6.3, 8.0 μg/mL; c1-4 after 72 h c1: control, c2-4: concentrations of 4, 5.4, 6 μg/mL

**Fig. 6:**
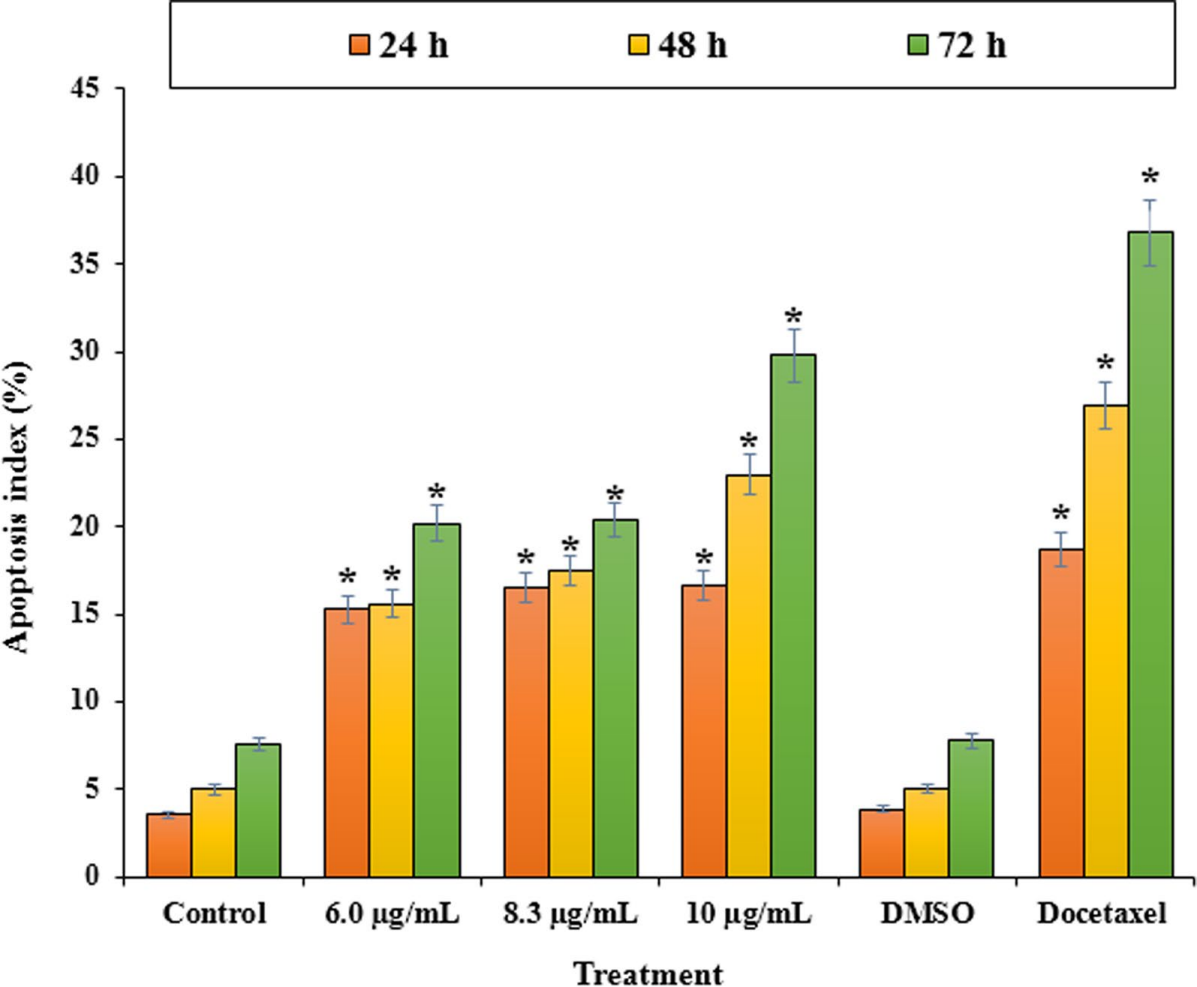
PI-staining induced apoptosis on LNCaP cells. LNCaP cells were treated with different concentrations of *L. usitatissimum* extract for 24, 48, and 72 h. *Indicate the significant difference (*p* < 0.01) between experimental groups with control/DMSO groups

**Fig. 7:**
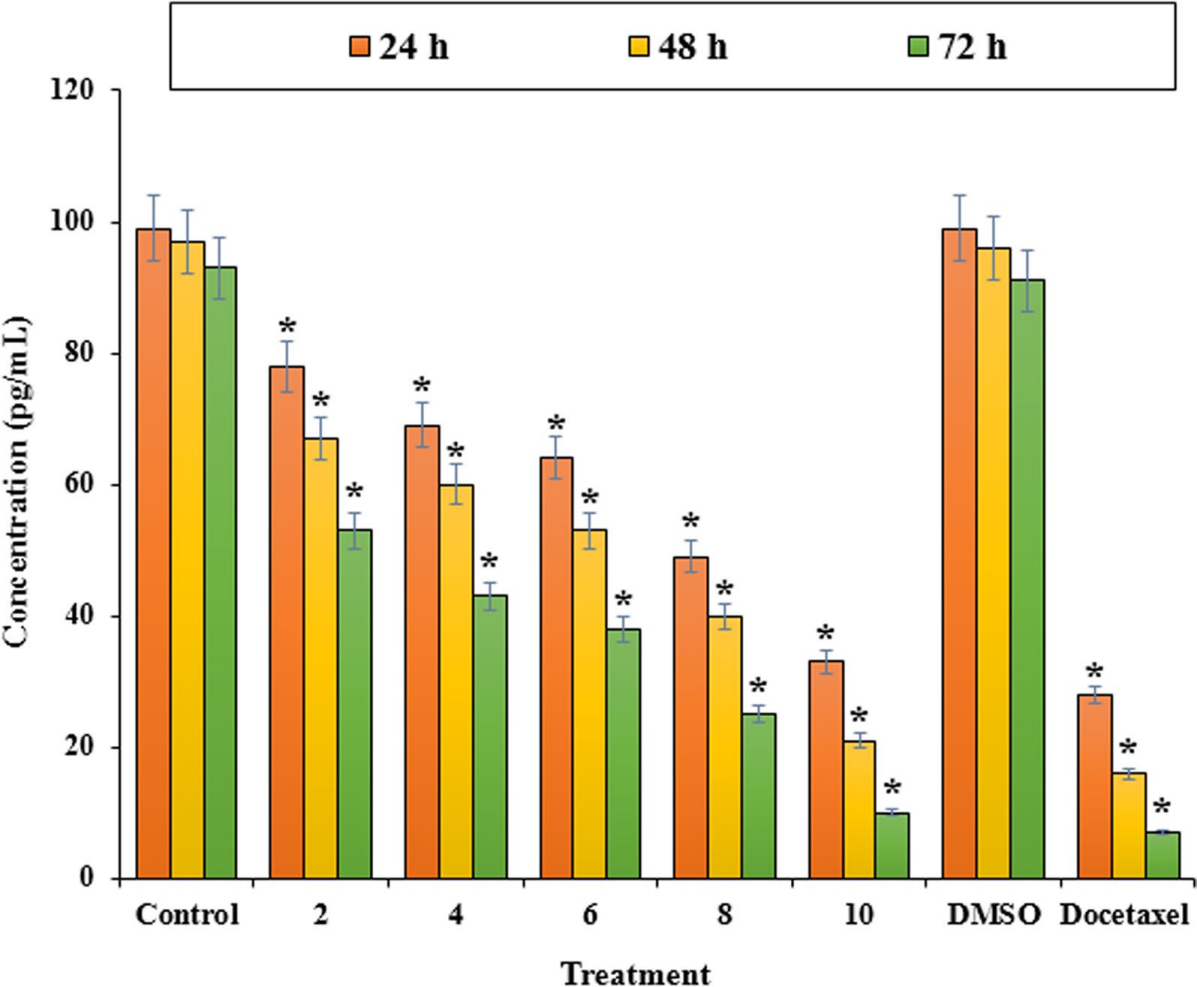
The effect of *L. usitatissimum* extract on the concentration of Tumor Necrosis Factor-Alpha (TNF-α) of LNCaP cells. The concentration was measured by the MTT assay. LNCaP cells were treated with different concentrations of the extract for 24, 48, and 72 h. *Indicate the significant difference (*p* < 0.01) between experimental groups with control/DMSO groups

Apoptosis or cell death is considered as an ordered cellular process. The physiological (such as programmed cell destruction, physiologic involution, and regular destruction of cells) and pathological (such as anticancer drug, progressive cell death, and pathologic atrophy of organs and tissues) conditions affect the apoptosis (Wong 2011). Since, resisting death and avoiding apoptosis is one of the ten cancer hallmarks (Hanahan and Weinberg 2011), a study on the apoptosis process is applying for an important role in oncology research (Jian et al. 2018). Figures 5 and 6 exhibit Flow cytometric evaluation and the apoptosis percentage by LUE that was quantitatively determined using PI staining. For this assay, LNCaP cells were treated by *L. usitatissimum extract* with three different concentrations (treatment 1: less than IC_50_, treatment 2: IC_50_, and treatment 3 more than IC_50_) that were obtained from MTT assay for 24, 48, 72 h. The treatment of LNCaP cells with LUE significantly increased the percentage of apoptosis as compared to the control. The highest apoptosis was observed for LUE with the concentration of 6.0 μg/mL after 72 h with the amount of 29.8%, and the smallest one belongs to the concentration of 6.0 μg/mL after 24 h. Figure 7 indicates the concentration of Tumor Necrosis Factor-Alpha (TNF-α) in several examined groups. The best results were seen in the highest dose of extract and Docetaxel.

### Bax and caspase3 gene expressions

The caspases are a family of protease enzymes. They have a critical role in the cell's apoptotic process (Hu et al. 2019). Caspases are usually activated in the early stages of apoptosis (Kilinc et al. 2020). These proteins affect significantly through activation of the death receptors and mitochondrial pathways. Caspase-3 is known as the primary executioner of the family that plays a vital function in reaching apoptotic cell death by cleaving the cellular substrates(Abou-Hashem et al. 2019). Bax (Bcl-2-associated X protein) is a tumor suppressor gene that can promote cell apoptosis (Ammoury et al. 2019; Liu et al. 2015). The expression level of Bax and caspase-3 is a known way to evaluated induced apoptosis mechanism (Deng et al. 2017).

**Fig. 8:**
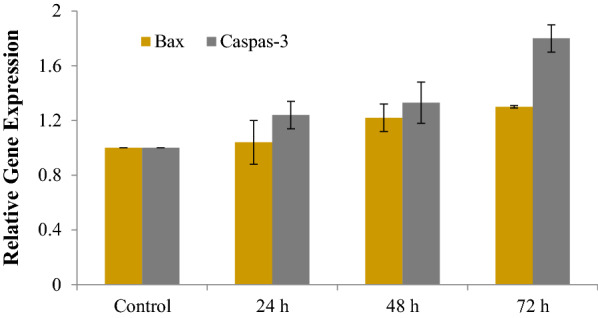
The effect of *L. usitatissimum* on the expression of caspase-3 and Bax ratio in LNCaP cells evaluated using the Real-Time PCR method. Cells were treated with the IC_50_ dose with the extract. The significant difference between control groups with Treatment group is indicated*;* *p < 0.05, **p < 0.01 and ***p < 0.001. IC_50_ doses at different times: 24 h: 8.3 μg/mL; 48 h: 6.3 μg/mL and 72 h: 5.4 μg/mL

Figure 8. presents Bax and caspase-3 gene expression of treated LNCaP cells by *L. usitatissimum* extract using the real-time qPCR assay. Based on the findings, the gene expression levels were correlated to the selected apoptosis-inducing factor (Bax and caspase-3) after 24, 48, and 72 h. According to the results, the genes had been up-regulated in the extracts-treated cells compared to those of control cells (*p < 0.05, **p < 0.01 and ***p < 0.001). The outputs revealed higher levels of expressions for Bax and caspase-3 in the extract –receiving treatments.

## Discussion

The previous studies on the HPLC analysis revealed the phenolic profile of *L. usitatissimum* extract. Han et al. (2018). Have reported the plant was rich in *p*-hydroxybenzoic acid *p*-coumaric acid. In another study, HPLC analysis showed sinapic and *p*-hydroxybenzoic acids were abundant compounds in *Linum usitatissimum* root extract (Gabr et al. 2018). Our result also exhibits the presence of sinapic and *p*-coumaric acid in LUE; however, the extract was rich in gallic acid ferulic, and vanillic acid.

Han et al. (2018) reported the ability of the ethanolic and aqueous extract of flaxseed shell to scavenge DPPH radical with 49.50 and 53.30 μg/mL that was less than LUE activity. On the other hand, the ability of their extract to chelate ferrous ions 9.24 and 8.88 μg/mL was more than the extract of this research.

The chemical constituents of *L. usitatissimum* extract contributed to the anticancer activity or induced apoptosis on LNCap cells. On the other hand, the synergic effect of the plants' compositions is a well-known factor behind the plants' role to cure a wide range of unhealthy problems (Mahdavi et al. 2017). According to the HPLC results and previous studies, mentioned in the introduction section, *L. usitatissimum* is rich in phenolic, flavonoids, and lignan compounds. The anticancer activity of these compounds was reported previously. For instance, gallic acid can be considered as an agent in the treatment of prostate cancer (Heidarian et al. 2016). Various reports have been found in the literature on the mechanisms of gallic acid effects on prostate cancer cells. For example, through a ROS-dependent apoptotic mechanism (Russell et al. 2012); through induction of mitochondrial apoptotic signaling pathways (Chen et al. 2009); DNA damage (Liu et al. 2013); reducing protein IL-6 and pAKT signaling protein pathways (Heidarian et al. 2017); increased p27 levels (Reddivari et al. 2010); promotes the levels of phosphatidylinositol 3-kinase (PI3K) and AKT in PC-3 cells (Liu et al. 2011). Ferulic acid is another abundant phenolic compound in the plant extract reported as an active agent against the proliferation of prostate cancer cells (Eitsuka et al. 2014; Eroğlu et al. 2015). Two research groups have reported that chlorogenic acid showed a restraining effect on benign prostatic hyperplasia (Huang et al. 2017; Yamagata et al. 2018). Hydroxycinnamic acids such as caffeic acid have a potential inhibitory effect on prostate cancer cell invasion and metastasis (Rocha et al. 2012). Overall, diets that are rich in natural phenolic compounds have valuable effects in reducing prostate cancer incidence (Russo et al. 2017). On the other hand, the synergistic action of phenolic compounds with chemotropic drugs has also been reported as an effective strategy in cancer treatment (Damasceno et al. 2017; Eroglu et al. 2018).

Polyphenols such as Lignans exhibit anticancer activity mainly against breast, prostate, and colon cancer (Zahir et al. 2019). Lignans prevent prostate cancer growth via numerous mechanisms of action (Yatkin et al. 2014). These molecules were the most effective as a death receptor-sensitizing agent (Peuhu et al. 2010).

Based on this research, gallic, ferulic, and vanillic acid were found as the abundant phenolic compounds in the hydroalcoholic extract of *L. usitatissimum* seeds (flaxseeds). The plant extract showed a high level of antioxidant activity to scavenge the free radical of DPPH with a low IC_50_, even less than BHT. *L. usitatissimum* extract exhibited a remarkable cytotoxicity effect on human prostate cancer cells of LNCaP. The values of 8.3, 6.3, and 5.4 μg/mL were obtained as IC_50_ for the treatment of the cell lines after 24, 48, and 72 h. The extract also induced apoptosis on the cells line with a minimum of 15.3% and a maximum of 29.8%. The gene expression of caspase-3 and Bax also increased after treatment of the cells with the plant extract. Based on our in *vitro* study with LNCaP cells, *L. usitatissimum* induces apoptotic cell death. As follow up to this, further studies are needed to evaluate the therapeutic potential of *L. usitatissimum* against PCa in experimental in *vivo* models.
